# Quercetin Inhibits Pyroptosis in Diabetic Cardiomyopathy through the Nrf2 Pathway

**DOI:** 10.1155/2022/9723632

**Published:** 2022-12-31

**Authors:** Zhang Wei, Zhou Jing, Kang Pinfang, Shi Chao, Qian Shaohuan

**Affiliations:** ^1^Department of Cardiovascular Medicine of The First Affiliated Hospital of Bengbu Medical College, Bengbu City, Anhui, China 233000; ^2^Department of Physiology of Bengbu Medical College, Bengbu City, Anhui, China 233000; ^3^Department of Cardiac Surgery of The First Affiliated Hospital of Bengbu Medical College, Bengbu City, Anhui, China 233000

## Abstract

The present study investigated whether quercetin promotes the nuclear translocation of nuclear factor erythroid-2-related factor 2 (Nrf2) to inhibit pyroptosis progression and ameliorate diabetic cardiomyopathy. We evaluated the protective effects of quercetin against diabetic cardiomyopathy by analyzing the expression of pyroptosis pathway proteins, myocardial cell apoptosis rate, degree of myocardial fibrosis, and serum inflammatory indices in the hearts of model rats with diabetes. We evaluated the expression of Nrf2 in the nucleus of cardiomyocytes and H9C2 cells to clarify the role of quercetin in promoting the nuclear translocation of Nrf2. In addition, we coincubated cardiomyocytes with the Nrf2 inhibitor ML385 to confirm that quercetin inhibits the diabetes-induced cardiomyocyte pyroptosis via the Nrf2 pathway. We found that quercetin promoted the nuclear translocation of Nrf2 in cardiac cells of diabetic rats, increased the expression of the antioxidant proteins HO-1, GCLC, and SOD, reduced the accumulation of ROS and the degree of cardiomyocyte apoptosis, and alleviated diabetes-induced cardiac fibrosis. The therapeutic effects of quercetin were further validated in H9C2 cardiomyocytes. Interestingly, ML385 prevented the beneficial effects of quercetin on diabetic cardiomyopathy, further indicating that the quercetin-mediated inhibition of pyroptosis requires the participation of the Nrf2 pathway. In conclusion, quercetin promoted the nuclear translocation of Nrf2, increased the expression of antioxidant factors in cells, and inhibited the progression of cell pyroptosis, thereby alleviating diabetic cardiomyopathy.

## 1. Background

Diabetic cardiomyopathy is a common complication of diabetes mellitus, associated with a potentially insidious early stage and late-stage complications, such as endothelial damage to the coronary vessels and decreased myocardial contractility [1]. Exposure of cardiac myocytes in a high-glucose state for an extended time period results in insulin resistance increased glucose autoxidation, protein glycosylation, lipid peroxidation, intracellular peroxide accumulation, cardiomyocyte death, and enhancement of cardiac fibrosis [2, 3].

Pyroptosis is a distinct caspase-dependent inflammatory cell necrosis characterized by cell lysis and the release of cytosolic contents into the surrounding environment, resulting in a strong inflammatory response [4, 5]. Various stimuli can induce pyroptosis in cells. Pi et al. discovered that molybdenum induced pyroptosis in renal tubular epithelial cells, whereas drugs that prevent peroxide accumulation significantly reduced the expression of NLR family pyrin domain containing 3 (NLRP3) and other molecules involved in the classic pyroptosis pathway, reducing epithelial cell damage [6]. In cardiovascular disease, molecules such as high fat and urea can induce the pyroptosis of cardiomyocytes, which triggers a cascade reaction and the release of a large number of inflammatory mediators, resulting in the development of a local inflammatory microenvironment in the myocardium [7, 8]. Therefore, preventing the development of pyroptosis can slow the progression of diabetic cardiomyopathy.

Quercetin is a natural flavonoid that is found in a wide range of plants. Previous studies have indicated that quercetin has antioxidant and anti-inflammatory properties. Tan et al. found that quercetin inhibited the macrophage-mediated inflammatory response by regulating the Mincle/Syk/NF-*κ*B signaling pathway, thus alleviating cisplatin-induced acute kidney injury [9]. Sul et al. demonstrated that quercetin prevented lipopolysaccharide- (LPS-) induced oxidative stress and inflammation by modulating the NOX2/ROS/NF-*κ*B pathway in lung epithelial cells [10]. NFE2 like bZIP transcription factor 2 (NFE2L2; also known as nuclear factor erythroid-2-related factor 2 (Nrf2)) is the primary regulator of cellular resistance to oxidative stress. When the body is subjected to oxidative stress, cellular Nrf2 translocates to the nucleus, where it promotes the expression of a wide range of antioxidant factors, thereby maintaining the redox balance of the body [11, 12]. To our knowledge, there have been no reports on whether quercetin can promote the nuclear translocation of Nrf2 and inhibit the progression of diabetes-induced pyroptosis. Therefore, in this study, we aimed to investigate this issue.

## 2. Materials and Methods

### 2.1. Construction of the Diabetic Rat Model

Two-month-old Sprague–Dawley male rats were purchased from the Bengbu Medical College Experimental Animal Center (Bengbu, China). The diabetic rat model was established by high-fat feeding for 4 weeks and intraperitoneal injection of streptozotocin (STZ, 50 mg/kg). The successful establishment of the model was confirmed when the level of fasting blood glucose was over 11 mmol/L on 2 serial measurements or over 14 mmol/L in a single evaluation. The rats were divided into the normal (CON-Group), diabetic (DM-Group), interval dosing+diabetic (Qod-Group), and daily dosing+diabetic (Qd-Group) groups (*n* = 6). The quercetin stock solution was prepared according to the ratio of 50 mg quercetin dissolved in 1 mL DMSO solution. In the treatment group, the rats were fed quercetin at a dosage of 160 mg.kg^−1^.d^−1^. According to the body weight of each rat, an appropriate amount of quercetin stock solution was mixed with 5 mL of drinking water and fed to the rats by gavage. In the case of untreated groups, a mixture of DMSO and drinking water without quercetin was used as a control. The rats in each group were housed for 6 months under the same conditions. All animals were raised in the Bengbu Medical College Animal Experiment Center, separated in different cages according to experimental groups. The experimental environment was managed by the Animal Center: a room temperature of 26°C and 12 h light-dark cycles were maintained, and the breeding environment was cleaned regularly by professionals. All experimental procedures were approved by the Medical Ethics Committee of the First Affiliated Hospital of Bengbu Medical College (approval no. BYYFY-2021KY03).

### 2.2. Myocardial Tissue Sections

Following anesthesia with ether, the rats were euthanized by cervical dislocation. Myocardial tissues (approximately 1 cm^2^ in size) were extracted from the apical part of the heart, fixed with 4% paraformaldehyde, and embedded in paraffin. Myocardial sections were stained with Masson's trichrome stain and hematoxylin and eosin (H&E) according to the respective manufacturer's instructions. All remaining animal carcasses were sent to the animal room for unified disposal.

### 2.3. Detection of Serum Indicators Using ELISA

Venous blood was drawn from the tail veins of the rats, and the supernatant was collected after centrifugation and stored at −80°C. The levels of serum inflammatory markers and indicators of myocardial damage, including NF-*κ*B, IL-1, TNF-*α*, and brain natriuretic peptide (BNP), were estimated by using respective ELISA kits; each serum sample was analyzed 6 times.

### 2.4. High-Sugar Treatment of Cardiomyocytes

Rat H9C2 cardiomyocytes were purchased from the Shanghai iCell Biological Company, China, and were stored and cultured in the Cardiovascular Laboratory of Bengbu Medical College. The cells were divided into a normal medium group (Con-G, with medium glucose concentration of 5.5 mmol/L) and a high-glucose medium group (H-G, with medium glucose concentration of 30 mmol/L).

### 2.5. Extraction of Nucleoprotein from Cardiomyocytes

Digest the cells with EDTA, collect the cells by centrifugation at 500 g for 3 minutes, discard the supernatant, and save the pellet for later use. 200 *μ*L cell plasma protein extraction reagent was added to the pellet according to the manufacturer's instructions and mixed well in an ice bath (4°C) for 10 min; the resulting mixture was centrifuged at 12 000 g for 15 min, and the supernatant was discarded. Subsequently, the cellular nucleoprotein extraction reagent was added to the pellet and mixed thoroughly in an ice bath (4°C) for 10 minutes; the mixture was centrifuged at 12 000 g for 15 min, and the supernatant, which represents the cellular nucleoproteins, was collected.

### 2.6. Measurement of Mitochondrial Membrane Potential

A membrane potential staining solution was created by adding 2 *μ*L JC-1 solution to 900 *μ*L JC-1 solvent and 100 *μ*L JC-1 staining buffer and mixing thoroughly. The media was aspired from cultured-treated cardiomyocytes and replaced with a mixture of staining solution and culture medium at a 1 : 1 ratio. Cells were incubated for 20 min at 37°C, the staining solution was washed away with PBS, and the mitochondrial membrane potential was observed under a fluorescence microscope.

### 2.7. TUNEL Assay

Cell slides or tissue frozen sections were fixed with paraformaldehyde and rinsed thrice with PBS. After 15 min of membrane permeabilization with 0.5% Triton X-100, slides were rinsed thrice with PBS; the prepared stain was dripped on their surface and incubated at 37.5°C for 1 h in the dark. After washing thrice with PBS, cells were counterstained with DAPI and observed under a microscope.

### 2.8. Main Reagents

The following reagents were purchased from the respective suppliers: nuclear protein extraction kit (R0050), ML385 (IM1020), quercetin (SQ8030) (Solarbio Life Science, Beijing, China); GCLC rabbit polyclonal antibody (AF6969), Nrf2 rabbit polyclonal antibody (AF7623), HO-1 rabbit polyclonal antibody, one step TUNEL apoptosis assay kit (C1086), reactive oxygen species assay kit (S0033s), enhanced mitochondrial membrane potential assay kit with JC-1 (C2003S), and cell counting kit-8 (C0037) (Beyotime Biotechnology, Shanghai, China); NLRP3 rabbit polyclonal antibody (GB13457) and caspase-1 rabbit polyclonal antibody (GB11383) (Servicebio, Wuhan, China); DMEM high-glucose medium (SH30003; Hyclone Laboratories LLC, Logan, UT, USA); mRNA primers (Tsingke Biotechnology, Nanjing, China); HE staining kit (G1001), and Masson's trichrome stain kit (GP1032) (Servicebio); BNP (SEKF-0205), TNF-*α* (SEKR-0009), IL-1(SEKR-0002), and NF-*κ*B (K009537P) (Solarbio Life Science).

### 2.9. Statistical Analyses

The SPSS software (version 25.0) was used to process all statistical data. Briefly, 6 duplicate wells were set for each serum sample in ELISA measurements, 6 duplicate wells were set for each group in the CCK-8 test, while western blot analyses were repeated thrice. We ensured that each set of data and not the results obtained from a single measurement was repeated more than 3 times. Quantitative data are presented as the mean ± SD; pairwise comparisons between data were performed using the *t*-test. For comparison between multiple groups of data, ANOVA data analysis was performed. Images were analyzed using the ImageJ software. Statistical significance was set at *P* < 0.05.

## 3. Results

### 3.1. Quercetin Ameliorated Myocardial Fibrosis in Diabetic Rats

**Figure 1:**
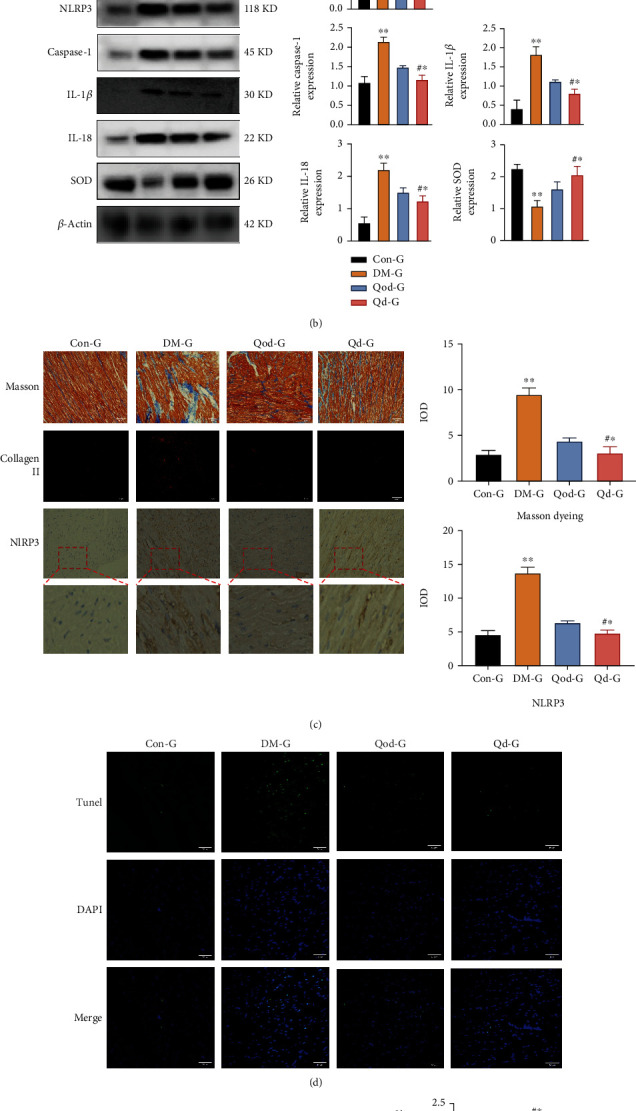
Quercetin ameliorates myocardial fibrosis in diabetic rats (a) ELISA assays for detecting the expression levels of NF-*κ*B, IL-1, TGF-*α*, and BNP in the serum of rats in each group. (b) Expression levels of pyroptosis pathway-associated proteins in the myocardial tissue of rats in each group detected by western blotting (WB). (c) H&E and Masson's trichrome staining of rat myocardial tissues, and immunofluorescence detection of the expression of collagen II in myocardial tissues (200× magnification). Quantitative analysis of the expression of collagen II in Masson's-stained sections and NLRP3 immunohistochemistry using ImageJ. (d) TUNEL assay detecting the apoptotic levels of myocardial tissue cells in each group (200× magnification). (e) Expression levels of Nrf2 in the nucleus and those of HO-1 and GCLC in the cell were detected by WB. H3: histone H3, used as a reference nuclear protein for normalization. CON-Group: control group; DM-Group: diabetic group; Qod-Group: interval dosing+diabetic group; Qd-Group: daily dosing and diabetic group; WB: western blotting. Data are expressed as the mean ± SD, ^∗∗^*P* < 0.001, DM-G vs. Con-G, and Qod-G. ^#^^∗^*P* < 0.001, Qod-G vs. Qd-G. ^^^^∗^*P* < 0.001, Qod-G vs. Con-G, and DM-G.

We used ELISA to identify and evaluate the levels of inflammatory markers and indicators of myocardial damage, including NF-*κ*B, IL-1, TNF-*α*, and BNP, in the serum of diabetic rats. We observed that administration of quercetin significantly reduced the levels of inflammatory factors in the serum of diabetic rats and decreased the secretion of BNP in the heart (Figure 1(a)). Importantly, we detected that STZ-induced diabetes increased the expression of pyroptosis proteins, whereas decreased that of superoxide dismutase (SOD) proteins in myocardial tissues (*P* < 0.001) (Figures 1(b) and 1(c)). However, quercetin significantly inhibited the progression of pyroptosis in the diabetic myocardium, with its therapeutic effect becoming more pronounced with the increase in the frequency of administration (*P* < 0.001)(Figures 1(b) and 1(c)). Histological analysis of tissue sections further indicated that quercetin reduced diabetes-induced cardiomyocyte apoptosis and inhibited the expression of collagen II and the progression of myocardial fibrosis (*P* < 0.001) (Figures 1(c) and 1(d)). In addition, we extracted nuclear proteins from the heart tissue and found that quercetin increased the expression and translocation of Nrf2 in cardiomyocyte nuclei (nu-Nrf2) and activated the expression of the downstream antioxidant factors, heme oxygenase-1 (HO-1) and recombinant glutamate cysteine ligase (GCLC) (Figure 1(e)). Therefore, we speculated that quercetin ameliorates diabetic myocardial injury via the Nrf2 pathway.

### 3.2. Quercetin Reduced High-Glucose-Induced Cardiomyocyte Injury

**Figure 2:**
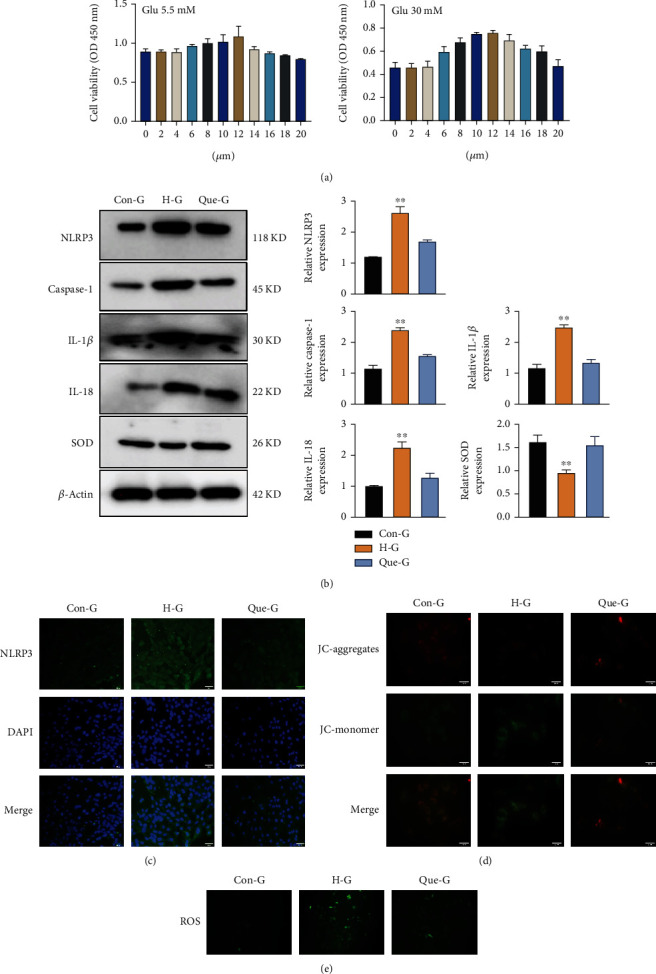
Quercetin ameliorates high-glucose-induced cardiomyocyte injury. (a) CCK-8 revealed changes in cell viability after coincubating H9C2 cells with different concentrations of quercetin for 24 h. (b) Expression levels of pyroptosis-related proteins detected by WB. Data are expressed as the mean ± SD, ^∗∗^*P* < 0.001, H-G vs. Con-G, and Que-G. (c) The expression level of NLRP3 in cardiomyocytes from each group detected by immunofluorescence (200× magnification). (d) Fluorescence detection of mitochondrial membrane potential. The brighter the red fluorescence, the stronger the mitochondrial membrane potential; the opposite condition stands for green fluorescence (200× magnification). (e) A ROS fluorescent probe was used to detect the accumulation of ROS in cardiomyocytes in each group (200× magnification). CON-G: glucose concentration of 5.5 mmol/L; H-G: glucose concentration of 30 mmol/L; Que-G: glucose concentration of 30 mmol/L+quercetin; ROS: reactive oxygen species.

To better understand the mechanism of action of quercetin in cardiomyocytes, we performed further analyses. According to the CCK-8 assay, the ideal dose of quercetin for increasing cardiomyocyte activity was 12 *μ*M (Figure 2(a)). We also found that quercetin dramatically reduced the high-glucose-induced expression of pyroptosis proteins in cells, whereas enhanced that of SOD proteins as indicated by western blot analysis, thus successfully suppressing pyroptosis (Figures 2(b) and 2(c)). In addition, we observed that quercetin reduced the accumulation of reactive oxygen species (ROS) in cardiomyocytes and ameliorated the mitochondrial damage caused by high glucose, as indicated by the evaluation of the mitochondrial membrane potential and intracellular level of reactive oxygen species in cardiomyocytes (Figures 2(d) and 2(e)).

### 3.3. Quercetin Promoted the Nuclear Translocation of Nrf2

**Figure 3:**
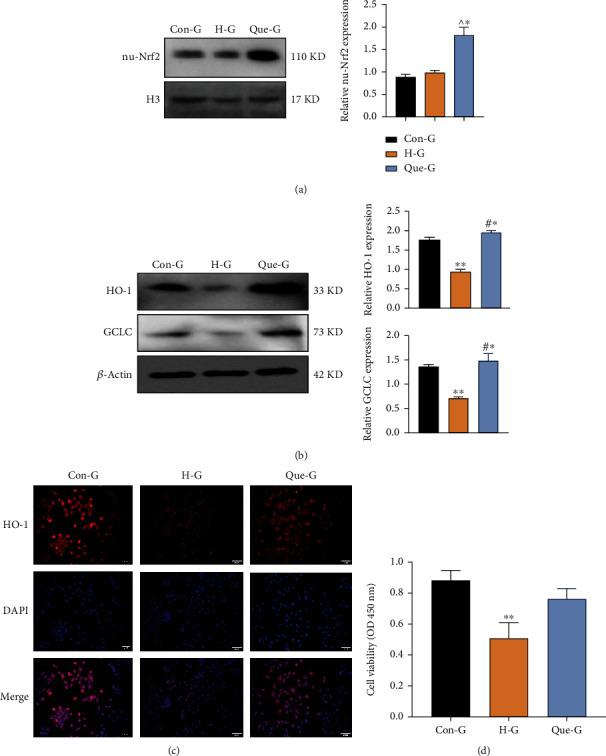
Quercetin promotes the nuclear translocation of Nrf2. (a) Expression levels of Nrf2 in the nucleus detected by WB. (b) Expression levels of HO-1 and GCLC detected by WB. (c) Immunofluorescence detection of the expression level of HO-1 in cells (200× magnification). (d) Viability of cells in each group detected by CCK-8 assay. H3: histone H3, used as a reference nuclear protein for normalization. CON-G: glucose concentration of 5.5 mmol/L; H-G: glucose concentration of 30 mmol/L; Que-G: glucose concentration of 30 mmol/L+quercetin; WB: western blotting. Data are expressed as the mean ± SD. ^∗∗^*P* < 0.001, H-G vs. Con-G and Que-G. ^#^^∗^*P* < 0.001, Que-G vs. Con-G. ^^^^∗^*P* < 0.001, Que-G vs. Con-G and H-G.

We extracted nuclear proteins from H9C2 cells and evaluated the expression of Nrf2 in the nucleus (nu-Nrf2). We found that quercetin significantly upregulated the expression and accelerated the nuclear translocation of Nrf2 in cardiomyocytes (*P* < 0.001) (Figure 3(a)). We also observed that the expression of the HO-1 and GCLC downstream antioxidant factors was also markedly elevated following the nuclear translocation of Nrf2 (Figures 3(b) and 3(c)). Accordingly, we detected that overexpression of these antioxidant proteins led to a significant increase in the viability of cardiomyocytes in high-glucose media (*P* < 0.001) (Figure 3(d)). These results suggested that the inhibitory effect of quercetin on pyroptosis is related to the activation of the Nrf2 pathway.

### 3.4. ML385 Inhibited the Nuclear Translocation of Nrf2

**Figure 4:**
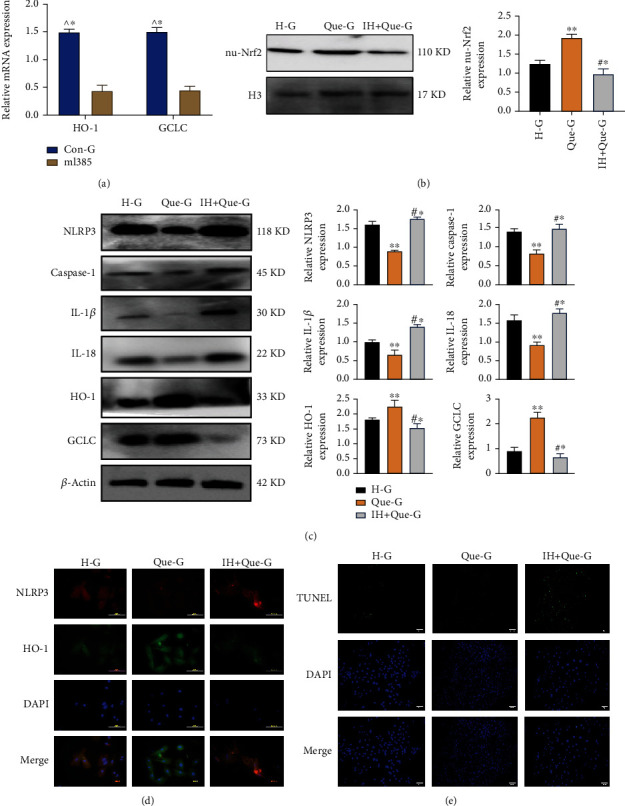
ML385 inhibits the nuclear translocation of Nrf2. (a) mRNA expression levels of HO-1 and GCLC detected by PCR. (b) Expression of Nrf2 in the nucleus detected by WB. (c) Expression levels of pyroptosis pathway-related proteins detected by WB. (d) Expression of NLRP3 and HO-1 in cells detected by immunofluorescence (200×). (e) A TUNEL fluorescent probe was used to detect the incidence of apoptosis in each group (200×). H-G: glucose concentration of 30 mmol/L; Que-G: glucose concentration of 30 mmol/L+quercetin; IH+Que-G: glucose concentration of 30 mmol/L+quercetin+ML385. Data are expressed as the mean ± SD. ^^^^∗^*P* < 0.001, Con-G vs. ML385. ^∗∗^*P* < 0.001, Que-G vs. H-G and IH+Que-G. ^#^^∗^*P* < 0.001, IH+Que-G vs. H-G.

As ML385 is known to effectively block the Nrf2 pathway, we treated cardiomyocytes with 20 *μ*M ML385 to determine whether quercetin inhibited the pyroptosis pathway by inhibiting Nrf2. We found that ML385 significantly inhibited the mRNA expression of the antioxidant factors HO-1 and GCLC in the Nrf2 pathway (*P* < 0.001) (Figure 4(a)). Further experiments confirmed that the addition of ML385 inhibited the effect of quercetin against pyroptosis (*P* < 0.001); in particular, it increased the expression levels of pyroptosis-associated proteins and decreased those of the antioxidant proteins HO-1 and GCLC (Figures 4(b) and 4(c)). Consequently, we confirmed that ML385 abolished the ability of quercetin to inhibit apoptosis under high-glucose conditions (Figure 4(d)). These results further demonstrated that the Nrf2 pathway is necessary for quercetin to prevent pyroptosis.

## 4. Discussion

Diabetes creates a high-glucose environment in the myocardium, triggering an increase in the uptake and oxidation of fatty acids by myocytes for the generation of ATP. However, *β*-oxidation does not fully metabolize long-chain fatty acids, causing lipid accumulation and lipotoxicity in cells [13–15]. High levels of sugar promote the accumulation of cholesterol, fatty acids, and other related molecules in the blood and activate inflammatory factors such as TNF, IL-6, and IL-1, resulting in the worsening of myocardial damage [16, 17]. Furthermore, these factors can activate the pyroptotic pathway, resulting in cell membrane rupture, release of numerous inflammatory factors, increased cardiac fibrosis, and the formation of a local inflammatory microenvironment [18, 19]. Therefore, inhibition of cardiomyocyte pyroptosis is a potentially effective way to treat diabetic cardiomyopathy. Our study confirmed that diabetes led to pyroptosis in rat cardiomyocytes and promoted an increase in the levels of the inflammatory factors NF-*κ*B, IL-1, and TNF-*α*. Of note, long-term inflammatory stimulation promotes cardiomyocyte apoptosis and increases the expression of collagen, thereby aggravating myocardial fibrosis. Moreover, the concomitant accumulation of inflammatory stimulation, cell edema leads to a reduction in cardiac function and increases the secretion of BNP. Following administration of quercetin, the expression of pyroptosis proteins in the myocardium was markedly decreased, whereas the levels of antioxidant proteins, such as SOD, were elevated. In addition, the incidence of diabetes-induced myocardial cell apoptosis was inhibited, and the progression of myocardial fibrosis was slowed. Moreover, the degree of quercetin-mediated inhibition of pyroptosis and fibrosis became more pronounced with the increase in the frequency of quercetin administration.

Various studies have shown that Nrf2 is an important antioxidant that exerts a protective effect on the myocardium [20, 21]. Following stimulation by exogenous and related factors, cytoplasmic Nrf2 dissociates from Keap1 and translocates into the nucleus where it binds to antioxidant response elements (AREs) to promote the expression of target genes [22, 23]. Interestingly, these AREs are typically found in the genes of HO-1, GCLC, GSH, and other similar proteins, which scavenge oxygen free radicals, thus protecting cells from oxidative damage [24]. Cen et al. discovered that mitoQ promoted the nuclear translocation of Nrf2, scavenged intracellular peroxides, and upregulated the expression of downstream target genes containing antioxidant response elements, such as HO-1 and quinone oxidoreductase, to reduce cell damage [25]. Recent studies have found that quercetin can play a therapeutic role in various diseases by regulating Nrf2. Zhao et al. found that quercetin plays a role in the treatment of ethanol-induced hepatic steatosis by regulating the PI3K/Keap1/Nrf2 signaling pathway [26]. Hsu et al. found that quercetin reduced the mitochondrial production of reactive oxygen species through the Nrf2-PGC1*α*-Sirt1 pathway, thereby reducing retinal damage caused by sodium iodate [27]. However, there is still a lack of studies investigating the role of quercetin in the treatment of diabetic cardiomyopathy through the Nrf2 pathway. In our study, we found that quercetin significantly reduced the accumulation of inflammatory factors and ameliorated the diabetes-induced fibrosis in the myocardium of diabetic rats. We also confirmed that quercetin promoted the nuclear transfer of Nrf2, reduced the diabetes-induced cardiomyocyte pyroptosis, thus protecting the myocardium.

On the other hand, we found that quercetin reduced the accumulation of ROS in cardiomyocytes by increasing the expression of HO-1, GCLC, and SOD, thus inhibiting the high-glucose-mediated cell damage. There is an inseparable relationship between mitochondrial damage, ROS accumulation, and pyroptotic responses. The soluble protein in mitochondria can be recognized by apoptosis peptidase activating factor 1 (APAF1), which promotes the formation of pyroptotic body and the activation of Caspase-1 protein [28]. Pyroptosis further promotes mitochondrial shrinkage and inactivation, as well as the accumulation of ROS in mitochondria [29]. Intracellular accumulation of ROS activates the pyroptotic pathway through the activation of inflammasomes such as NLRP3 [30]. The interaction between ROS and pyroptosis amplifies the inflammatory response, leading to cell apoptosis. Therefore, improving the mitochondrial function and reducing the production of ROS can effectively alleviate pyroptosis. In the present study, we found that quercetin can inhibit the activation of pyroptotic bodies and mitochondrial damage induced by high glucose through the Nrf2 pathway, thereby improving diabetic cardiomyocyte injury. However, the efficacy of quercetin in preventing pyroptosis was considerably diminished after administration of ML385. These data suggested that quercetin can inhibit the progression of pyroptosis through the Nrf2 pathway, exerting a protective effect on diabetic myocardium.

## 5. Conclusion

Diabetic cardiomyopathy is a common complication in patients with diabetes. The mechanism underlying diabetic cardiomyopathy remains unknown, and till date, no specific therapeutic drugs for treating this condition are available. The present study found that quercetin promoted the nuclear translocation of Nrf2, increased the expression levels of the downstream antioxidant factors HO-1 and GCLC, cleared the accumulation of intracellular ROS, and inhibited the process of pyroptosis in diabetic cardiomyopathy. Thus, quercetin can reduce the stimulation of inflammatory factors in myocardial tissue, delay the development of myocardial fibrosis, and safeguard the function of the heart. This study elucidated the pathogenesis of diabetic cardiomyopathy and provided a new direction for the treatment of diabetes-associated disorders.

## Data Availability

The raw data supporting the conclusions of this article will be made available by the authors, without undue reservation.
